# Wildtype and A30P Mutant Alpha-Synuclein Form Different Fibril Structures

**DOI:** 10.1371/journal.pone.0067713

**Published:** 2013-07-04

**Authors:** Søren Bang Nielsen, Francesca Macchi, Samuele Raccosta, Annette Eva Langkilde, Lise Giehm, Anders Kyrsting, Anna Sigrid Pii Svane, Mauro Manno, Gunna Christiansen, Niels Christian Nielsen, Lene Oddershede, Bente Vestergaard, Daniel Erik Otzen

**Affiliations:** 1 Center for Insoluble Protein Structures (inSPIN), Interdisciplinary Nanoscience Center (iNANO) and Department of Molecular Biology and Genetics, Aarhus University, Aarhus, Denmark; 2 Department of Drug Design and Pharmacology, University of Copenhagen, Copenhagen, Denmark; 3 Institute of Biophysics, National Research Council of Italy, Palermo, Italy; 4 Niels Bohr Institute, Copenhagen University, Copenhagen, Denmark; 5 Center for Insoluble Protein Structures (inSPIN), Interdisciplinary Nanoscience Center (iNANO) and Department of Chemistry, Aarhus University, Aarhus, Denmark; 6 Department of Biomedicine, The Bartholin Building, University of Aarhus, Aarhus, Denmark; Hertie Institute for Clinical Brain Research and German Center for Neurodegenerative Diseases, Germany

## Abstract

Parkinson’s Disease (PD) is a neurodegenerative movement disorder affecting millions of people worldwide. One of the key players in the development of the disease is the protein α-synuclein (aSN), which aggregates in the brain of PD patients. The aSN mutant A30P has been reported to cause early-onset familial PD and shows different aggregation behavior compared to wt aSN. Here we use a multidisciplinary approach to compare the aggregation process of wt and A30P aSN. In agreement with previous studies, we observe an initial lag phase followed by a continuous structural development of fibrils until reaching an apparent monomer-aggregate equilibrium state and a plateau in Thioflavin T (ThT) fluorescence intensity. However, at later timepoints A30P shows greater propensity than αSN wt to form dense bundled fibril networks. Combining small angle x-ray scattering, x-ray fibre diffraction and linear dichroism, we demonstrate that while the microscopic structure of the individual fibril essentially remains constant throughout the experiment, the formation of dense A30P fibril networks occur through a continuous assembly pathway while the formation of less dense wt fibril networks with fewer contact points follows a continuous path during the elongation phase and a second rearrangement phase after reaching the ThT fluorescence plateau. Our work thus highlights that structural rearrangements proceed beyond the plateau in ThT-based monitoring of the fibrillation process, and the density and morphology of the resulting fibril networks is highly dependent on the aSN form studied.

## Introduction

Parkinson’s disease (PD) is a neurodegenerative movement disorder, characterized by the gradual loss of dopaminergic neurons and accumulation of aggregated protein deposits, known as Lewy bodies and Lewy neurites [1]. The major component of these deposits is the protein α-synuclein (aSN) [2] which is enriched in presynaptic terminals. Although the aggregation of aSN into amyloid fibrils characterized by classical cross-β structure [3] is considered to be of fundamental importance in the development of the disease, intermediate fibrillation species have been suggested to be the principal pathogenic species responsible for neuronal cell death [4]. In addition to sporadic onset of PD, familial forms of the disease such as the A30P [5] and A53T [6] mutations modulate the fibrillation pathway [7] and lead to early onset PD.

aSN is a 140 residues long natively unfolded protein [8], which upon incubation *in vitro* at 37°C with agitation can form fibrils resembling those extracted from PD patients [9], [10]. The disease-related A30P mutant is also natively unfolded, but sequence analysis of A30P based on a hierarchical neural method revealed a slightly increased tendency towards β-sheet formation, which could increase its aggregation propensity [11].

In contrast, NMR studies revealed higher overall flexibility of monomeric A30P [12] and A53T [12], [13] familial mutants in solution, suggesting a lower degree of shielding of the highly amyloidogenic NAC region and a higher level of hydration which has been suggested to increase the exposure of the NAC region and facilitate self-association [12].

Although the fibrillation core (residues 38–95) of wt and A30P mutant aSN has been shown to be conserved [14] and that the resulting fibrils formed by Wt and A30P aSN have been demonstrated to be predominantly straight (with some twisted fibres also observed for Wt) [15], a slightly increased fibril diameter of the A30P mutant (12±1.4 vs. 13±1.4 nm) [15] and a more polar and/or hydrated microenvironment in A30P samples attributed to the presence of internal cavities or water accessible areas surrounding A30P fibrils imply subtle conformational differences. Other mutations including but not limited to the familial A53T and E46K mutants have further been shown to produce fibrils with distinct morphologies [15], [16], indicating that the type and position of mutations govern fibril assembly and the resulting morphology.

The fibrillation kinetics of the A30P mutant has been shown to be slower than wildtype at moderate concentrations (∼1 mg/ml) [7], [11], [17], [18] but faster at higher (7 mg/ml) concentrations [19]. However, light scattering measurements [11], [18] and atomic force microscopy [20] have shown that αSN oligomers (defined as both fibrillar and non-fibrillar) species form faster for A30P than wt aSN prior to the emergence of fibrils. This may be important for early onset PD since cytotoxicity is believed to be linked with oligomeric species [21]. A recent Small Angle X-ray Scattering (SAXS) study on wt aSN revealed the low resolution solution structure of a wreath-shaped oligomer, formed during the fibrillation process, which is able to disrupt vesicles [22], while the structure of A30P oligomers remains unknown, apart from the shape of an isolated oligomer, revealed by transmission electron microscopy [17].

Despite significant recent advances, the mechanism of aSN aggregation and toxicity is still not understood in detail. Comparison of the molecular properties of fibrils from wt and familial mutant αSN may shed light on the phenomena causing the protein to aggregate into its toxic forms.

Here we present how a combination of several biophysical techniques reveals subtle differences in the molecular properties of wt and A30P aSN samples during fibrillation. We show how the two protein forms develop different molecular properties during the growth and elongation phases; importantly, we also observe significant structural rearrangements after reaching a plateau in terms of overall amyloid concentration (the latter measured by the fluorescent probe Thioflavin T). This plateau is often considered as the completion of the fibrillation reaction, but our data reveal that fibrils of the two proteins subsequently undergo significant but different rearrangements.

## Materials and Methods

### Expression and Purification of aSN

Wildtype aSN was recombinantly expressed in E. coli PSM1205 (Bioneer A/S, Aarhus, Denmark, kindly provided by Bjørn Holst) and A30P αSN was expressed in E. coli BL21 (DE3) cells carrying the pET11-D plasmid containing the aSN A30P gene. The recombinant proteins were expressed and purified according to ref [23] with an additional acid precipitation step. In brief, cells were harvested and subjected to osmotic shock treatment and adjusted to pH 3.5 for 20 minutes using HCl, leading to precipitation of the majority of most contaminating E. coli proteins, followed by centrifugation at 9000×g for 20 minutes and neutralization of the supernatant containing aSN to pH 7.5 using NaOH. SDS-PAGE showed no evidence of acid hydrolysis (data not shown). The protein was then purified by ion exchange chromatography, dialyzed exhaustively against MQ Water, lyophilized and stored at −20°C until further use as described [23].

### Fibrillation Assay

12 mg/ml solutions of both wt and A30P aSN were prepared by dissolving lyophilized protein in PBS buffer (pH 7.4) also containing 40 µM ThT followed by filtration through a 0.2 µm filter. The protein concentration was determined by UV absorbance at 280 nm using a NanoDrop (ND-1000, Thermo Scientific) and a calculated extinction coefficient of 0.412 mg cm^−1^ml^−1^.

150 µl aliquots were transferred to a 96 clear bottom wells plates for fluorescence measurements (Nunc, Thermo Fischer Scientific, Roskilde, Denmark) also including a 3 mm diameter glass sphere (Glaswarenfabrik Karl Hecht GmbH & Co KG). The plate was subsequently sealed with tape (Nunc, Thermo Fischer Scientific, Roskilde, Denmark) to avoid evaporation and incubated at 37°C in a Tecan GeniosPro fluorescence plate reader. ThT fluorescence intensities were measured at 15 min intervals (excitation wavelength 448 nm, emission 485 nm) and samples were shaken for 5 minutes with 300 rpm orbital shaking during each interval. At each time point, samples were collected by emptying the entire content of a well.

### Linear Dichroism

Linear Dichroism (LD) measurements were performed on a Jasco J-810 spectrometer (Tokio, Japan) adapted with a microvolume Couette flow cell (Dioptica, Rugby, UK). The solution used for the LD studies was prepared immediately after the sample collection from the plate reader, flash-frozen and kept in the freezer until the measurements were performed. Samples from the fibrillation assay were diluted to 0.1 mg/ml aSN and transferred to a Suprasil quartz capillary with inserted rod (Hilgenberg GmbH, Germany). Each sample was measured by performing a non-rotating scan, followed by 2 rotating scans and by a second non-rotating scan. To obtain a LD signal, the sample has to be macroscopically oriented: in our experimental setup. This was obtained by acquiring wavelength scans under rotation in a Couette flow cell. Non-rotating scans, in which the fibres are randomly oriented, were acquired as baselines. As fibrillation progressed, it became necessary for each time point to record several rotating scans until a stable LD signal was obtained. The last two stable rotating scans were then averaged to enhance the data quality and the average of the two baseline scans was subtracted.

The LD signal (measured in differential OD units, dOD) is the difference in absorbance of plane polarized light in parallel (A_∥_) and perpendicular (A_⊥_) to the direction of orientation [24]:
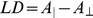
(1)


### SAXS

Data were collected at beamline X33 at the European Molecular Biology Laboratory on DORIS III (DESY) at a wavelength of 1.5 Å, using a MAR345 Image Plate detector, in the momentum transfer range 0.006< s <0.498 Å^−1^ (s = 4π sin θ/λ, where 2θ is the scattering angle). Measurements were from samples extracted directly from the 96-well plates while performing ThT fluorescence measurements, as described for wt protein [10] Samples were loaded manually into the exposure chamber and exposed for 2 min. Repeated exposure showed no detectable radiation damage and buffer scattering data were recorded before and after each protein scattering measurement. Raw 2D data were radially averaged and corrected for the detector response scaled to the incoming X-ray intensity. Data analysis was performed in ATSAS 2.1 [25] and in GRACE 5.1 (open source software).

The scattering curve from buffer at each time point *t* was obtained by averaging the two buffer scattering curves F(s, t), measured immediately prior to and immediately after each protein sample. We observed minute spread in buffer data, which may be ascribed to small variations in *e.g.* the accuracy in monitoring of the incoming beam intensity, X-ray alignment, perfection of the estimate of detector response and similar parameters related to the experimental devices. To compensate for this, we applied the simplest possible preliminary correction as follows: for each time point t, we identified two parameters, a’(t) and b’(t), which allowed us to transform F(s,t) into F(s,0) in the full q-range:
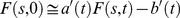
(2)


On a log-log scale, a′(t) and b′(t) correct for the shift in intensity and change in slope, respectively. Where different pairs of a′(t) and b′(t) values allowed the correction, the pair which minimizes their own individual values was chosen. Assuming that these fluctuations in buffer data have an instrumental origin, we applied the same corrections to corresponding protein scattering curves, which were measured at the same time as the averaged buffer signal.

Both before and after this initial minor correction, the resulting scattering data from protein samples showed a systematic trend at high s-values: the scattering at high angles decreases with time, even below the scattering observed at high angles for the corresponding buffer measurements. All protein samples were measured at identical protein concentrations and in identical buffers. We would expect scattering at high angles to be similar for all measurements, since high angle data are a result of small distances between protein and solvent, *i.e.* close to the surface of the protein, whereas scattering at lower angles will change dramatically, given the structural changes that take place during fibrillation. However, since we observe a systematic variation in high-angle scattering, we conclude that the fibril solutions exhibit a non-standard solution behavior. Since the buffer background stability has already been closely monitored and confirmed, these changes can only derive from actual structural changes in the analyzed samples. This can either be ascribed to changes in the solvent or solute. We analyzed this behavior by calculating the variant deviation of the protein data (*i.e.* the variation with which the high-angle scattering deviates from the expected stable scattering at high angles), thereby obtaining a new set of a and b values (different from the a’(t) and b’(t) values from eq. 2):
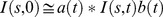
(3)


These a and b values were determined using the high s-range data only (2.34–4.5 nm^−1^). Here I(s,t) and I(s,0) represent the data before and after correction. Setting a(t) to 1.0 at t = 0, a(t) was confined to be close to 1 within the range 0.8–1.2, while b(t) is confined to be positive and start from zero.

After the correction using the appropriate time-dependent values of a and b from eq. 3, we obtained scattering data from protein samples optimally superimposed at high s-range and subtracted the buffer data from these scattering curves. SAXS data were thus ready for further analysis. Determination of a’(t), b’(t), a(t), b(t) and buffer subtraction was performed by GRACE 5.1.

The average molecular masses of solutes were estimated from the extrapolated relative forward scattering, I(0) using the Guinier approximat ion [26]. P(r) functions were estimated using the indirect Fourier transformation program GNOM [27], included in the ATSAS package.

### X-ray Fibre Diffraction of αSN Fibrils

Samples were prepared by centrifugation of 50 µl 12 mg/ml aSN sample extracted (at the following time points: 7.5 h; 10 h; 24 h; 48 h; 144 h) from the 96-well plates (following the ThT fluorescence development as for all other experiments performed) at 13000 rpm for 15 min to isolate and partially align fibrils while discarding the supernatant. The pellet was dried in a stream of nitrogen gas, a piece of the pellet was then mounted on a pin and placed in the beam.

X-ray diffraction data were collected at MAX-lab beamline 911-2 (Lund, Sweden) using a fixed wavelength of 1.04 Å with a thermostatted cryojet stream of nitrogen stream set at 4°C to reduce radiation damage to the sample. The sample to detector distance was 220 mm and data was collected on a MarCCD 165 detector using an exposure time of 60 seconds at multiple sample orientations. Radial averaging of the fibre diffraction data was performed using the program Clearer [28]. Radial averaging was done in slices of 60 degrees around the meridional and equatorial directions. The forward and reverse contributions (from the beam centre) were averaged and plotted (only data from 144 h are shown).

### TEM

5 µL of aSN fibril solution diluted to 2.4 mg/ml was applied to the nickel grid for 30 sec. The grids were washed with one drop of double distilled water and stained with 3 drops of 1% phosphotungstic acid pH 6.9 and blotted dry. Electron microscopy was done using a JEOL 1010 TEM at 60 kW. Images were taken using an Olympus KeenView camera. For size determination, a standard grid-size replica plate (2160 lines/mm) was used. The samples were flash-frozen right after the collection from the plate reader to avoid further development in the samples before analysis.

### Residual Soluble Protein Concentration

For the determination of the residual soluble protein concentration, 200 µl of pooled sample from 3 wells the fibrillation assay were spun down at 13000 rpm for 15 min. The protein concentration in the supernatant was measured three times for each sample by Nanodrop 1000 spectrophotometer (Thermo Scientific) and presented as the average ± standard deviation.

### NMR

To determine the amount of bound vs. free water in our system over time, a fibrillation assay was performed as described above, but including 5% D_2_O as internal calibrant in the samples from the start of the fibrillation reaction. Fibrillation time profiles monitored by ThT fluorescence indicated no effect of 5% D2O on the fibrillation process for either wt or A30P αSN (data not shown). At appropriate time points, samples were collected and 200 µl of pooled sample transferred to 3 mm thick NMR tubes (Bruker) and immediately flash-frozen in liquid nitrogen. Samples were thawed and measured in triplicates one by one. Experiments were done on a Bruker Avance-III 500 spectrometer (500.13 MHz) using a standard inverse triple-resonance TXI 5-mm probe. Using the equipped Bruker SampleJet, the 3 mm NMR tubes were loaded into the magnet. Samples were measured in random order to eliminate systematic errors. Experiments were single scan CPMGs in a 2D arrayed with number of cycles. The delay time (d20) was 500 µs for a 2τ of 1 ms. The 2D array was phased with respect to the first increment, the center of the peak of interest was picked and an integral region around the signal was chosen. Using the Topspin built-in T_1_/T_2_ analysis tool, the peak and integral region were used for fitting T_2_ as single exponential decays.

### Confocal Microscopy

12 mg/ml wt and A30P aSN samples were prepared in 150 mM PBS with 40 µM ThT filtered through 0.2 µm microfilters to volumes of 500 µL in glass vials with a small glass bead each. These were then incubated at 37°C at 100 RPM on a linear shaker with 10 µL samples taken at set time intervals. The products were diluted to 0.2 mg/ml into buffer onto a freshly plasma-etched cover glass. The wt and A30P aSN fibril structures were then imaged by a Leica SP5 confocal microscope, using a 458 nm laser line for excitation and a detection window of 468–600 nm. Cover glass slides were matched to the 1.2 NA water immersion objective by Leica (No. 11506279). Images acquired at the glass surface showed that image acquisition had essentially no effect on fibril imaging (data not shown).

## Results

### Kinetics of wt and A30P Fibrillation Suggest Subtle Differences in Fibril Assembly

To characterize the overall mechanism of aggregation, the fibrillation of aSN A30P and wt was followed by Thioflavin T (ThT) fluorescence using optimized fibrillation conditions adopted from [10]. This approach uses a glass bead and shaking to reduce variability between samples.

**Figure 1:**
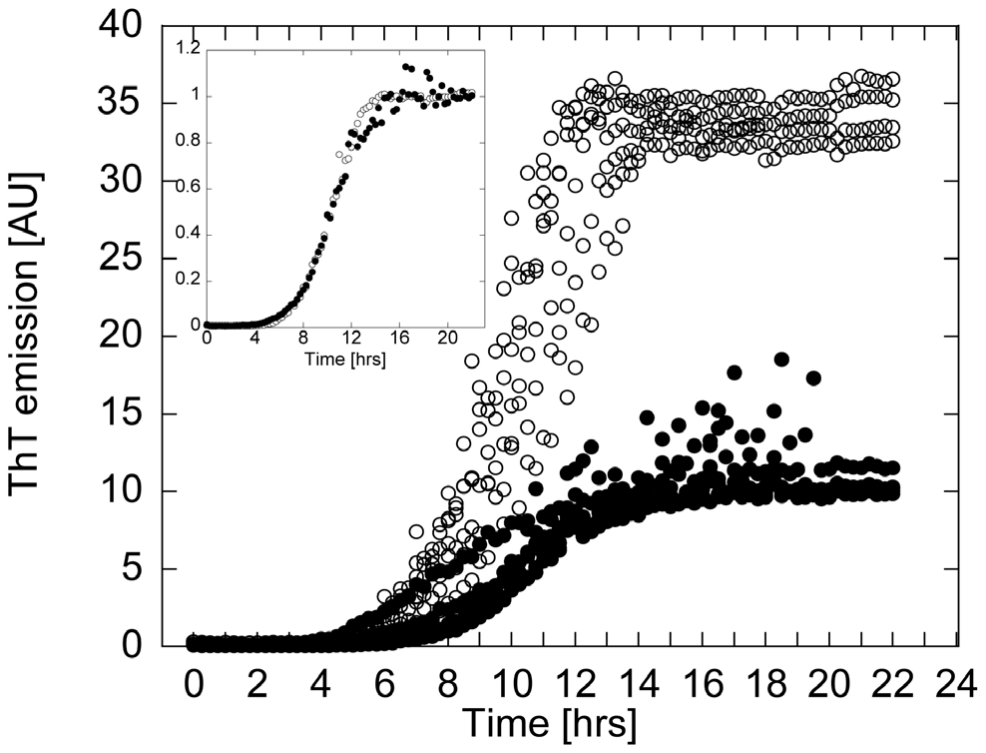
Kinetic profiles for wt (open symbols) and A30P (filled symbols) aSN fibrillation above the SCC. Insert: averaged and normalized ThT data show that wt and A30P aSN fibrillate with the same lag time and rate constant.

Kinetic traces for wt and A30P aSN both followed a sigmoidal course with a lag phase of around 5 h and an elongation phase until approximately 12 h followed by a plateau of essentially constant ThT fluorescence intensities (Fig. 1). Although identical concentrations of A30P and wt αSN were used, the absolute ThT intensity of A30P aSN samples was only ∼30% of the wildtype aSN signal. ThT fluorescence intensities of A30P and wt aSN normalized by the average fluorescence values at the plateau were largely superimposable (Fig. 1 insert), although elongation initially is most rapid for A30P. There were only minor differences in the later elongation phase, where A30P reached the plateau ∼3 h later than wt due to a slowly diminishing rate of elongation relative to wt aSN. The fluorescence readings around the final fluorescence values for A30P aSN fluctuate more than for wt samples, possibly due to the early formation of a more extensive fibril network for A30P than for wt aSN (see below). Dilution of A30P and wt aSN fibrils into buffer with and without fresh ThT revealed that the fluorescence intensity was not affected by the addition of fresh ThT (data not shown). This confirms that the ThT plateau was not caused by depletion of the ThT present in solution.

**Figure 2:**
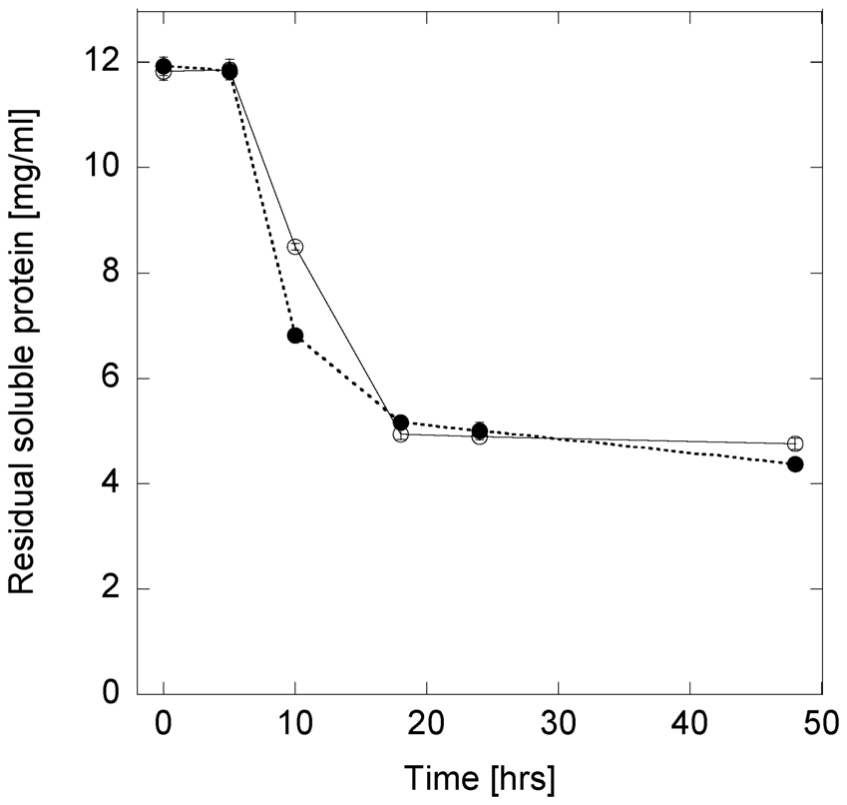
Residual protein concentration during the fibrillation of wt (open symbols) and A30P (filled symbols). The data represent the average of three replicates and the error bars their standard deviation. The depletion of soluble protein in the supernatant indicates the formation of large species.

The concentration of residual soluble aSN species dropped steeply after 5 h of incubation, coinciding with the increase in ThT fluorescence (Fig. 2). Soluble A30P levels dropped slightly more rapidly in the early elongation phase than wt levels, suggesting an initial rapid formation of fibrillated material. This agrees with A30P’s slightly higher initial rates of ThT-incorporation (Fig. 1). A plateau level of residual soluble aSN was reached after ∼18 h, similar to the ThT fluorescence time course. At this stage, both wt and A30P contain 4–5 mg/ml of soluble aSN left in solution. Thus wt and A30P form similar levels of fibrils, and both leave a significant amount (30–40%) of non-fibrillated material in solution.

### SAXS Data Clearly Reveal Late Protein Specific Fibril Rearrangements

**Figure 3:**
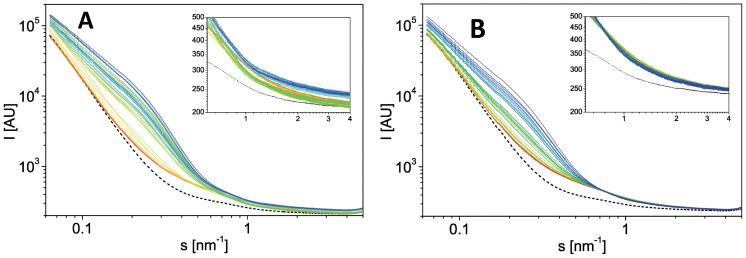
SAXS data from the protein samples during fibrillation. Data are for (A) aSN wt and (B) A30P. Intensity (arbitrary scale) is plotted as a function of the scattering vector s. Scattering curves are shown before the subtraction of the scattering from the buffer (shown in black) and the colouring changes from red to blue over time (0–24 h approximately). From the scattering curves, it is evident that intensity increases dramatically at low angles, due to the appearance of fibrils in the solution. Inserts zoom on the high s-values, emphasizing the surprising systematic changes of the intensities of scattering for the protein samples.

**Figure 4:**
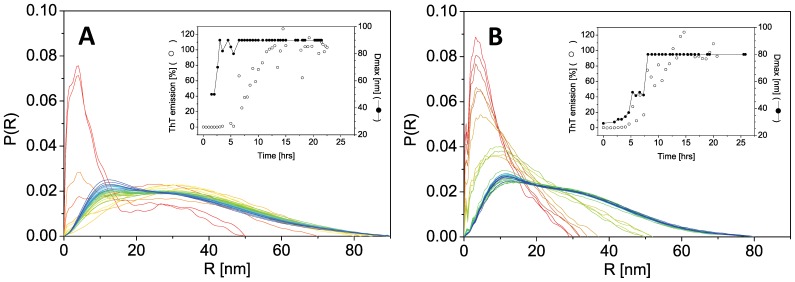
Pair distance distribution functions for the protein samples during fibrillation. Data are for (A) aSN wt and (B) A30P. Colours are as in Fig. 3. Inserts depict the development in D_max_, (filled symbols) versus time, plotted together with the development in the ThT signal (open symbols). It is evident that the maximally occurring D_max_ is present already at early time-points in both wt and mutant protein fibrillation. Likewise it can be seen that there is an increase in the observed D_max_ before an onset of fibrillation is registered via increas in the ThT intensity. The pair distance distribution functions are normalized to the same area under the curve, for better visualization of small species. The insert shows an increase in the maximum pair distance (filled symbols) prior to observed increases in ThT fluorescence (open symbols).

We used SAXS to compare the fibrillation of wt and A30P aSN. SAXS data were acquired on samples removed from the plate reader every ∼30 min. The raw data (Fig. 3AB) are converted into pair-distance distribution functions in Fig. 4AB which clearly reveal the development of large species in solution as a function of time. We previously reported that larger species of wt αSN form in solution before any changes are monitored by ThT fluorescence [22]. This is also seen for A30P. Fig. 4B (insert) shows that the calculated maximal pair distances (the maximal size within the scattering particles in solution) start to increase before the ThT-values and indicate formation of non-ThT-binding oligomers by both wt and A30P proteins. The pair distance distribution functions suggest a global maximum pair distance within the A30P αSN species in solution of ∼30 nm already after 2 h of fibrillation (Fig. 4B, insert). After ∼8 h and later in the fibrillation, an apparent maximum pair distance of ∼80 nm is observed. Over time even larger species are formed as indicated by the increase in the SAXS signal at low s values in Fig 3AB (due to the reciprocal relationship between s-values and scattering distances, low s values report on large scattering distances). We note that the maximal pair distance of ∼80 nm is a rough estimate, since this distance is beyond the detection limit of the scattering experiments. In accordance with previously published results [22], this maximal pair distance would be within the repeating unit of fibrils.

In addition to the expected increase in intensity at low angles (low s values in Fig. 3) as a result of the fibrillation process, an anomalous behavior was detected at higher scattering angles (higher s values in Fig. 3, insert). While no or minute changes are observed at high scattering angles in the repeated buffer scattering patterns, the changes in the protein samples are systematic and significant and hence relate to changes in the protein samples, and not to inaccuracies in the data. These higher s values relate to very small pair distances (<10 Å), and the scattering at these angles decreases significantly with time. This behavior is unexpected for the following reason. Since all the protein samples that were measured during this SAXS experiment were extracted from the same protein batch, prior to fibrillation onset, we expect identical protein concentration and identical buffer composition in all the samples measured. We expect the protein species to change shape during fibrillation, while different structures in the fibrillation pathway are formed. This is expected to be particularly visible at low scattering angles, which probe the long pair distances that are present in a given solution. Only pair distances where there is a contrast in the mean scattering density will result in scattering. For a two-phase system (protein and solvent), this contrast would be between the average protein scattering length density, and the average solute scattering length density. At high angles, these distances hence primarily relate to atom pairs between the surface of the protein and the solvent. When the protein fibrillates, larger species are formed in solution, resulting in a very different distribution of the long distances (protein-solvent distances), hence scattering at low angles changes dramatically. However, the pair-distances close to the surface should not change. A constant scattering is thus expected at high angles.

To elucidate this anomalous behavior, the data were corrected through a linear correction using eq. 3 (Materials and Methods). In this equation, the parameters a(t) and b(t) quantify the abnormal behavior of the fibrillating protein samples, compared to non-fibrillating samples. We interpret these parameters as follows: the multiplication factor a(t) depends upon the sample concentration as well as the scattering contrast between the solute and solvent. Given that the sample concentration is constant, changes in a(t) must be associated with changes in scattering contrast. The b(t) parameter is related to solvent scattering density alone and changes in b(t) are hence related to changes in solvent *properties* only. At the onset of fibrillation *(t = 0)*, water is in the normal liquid state and positive b values at time t mean that the corresponding sample at time t in total scatters less than water. This is possible if the solvent has a higher average density and/or lower compressibility than the sample [29], [30].

**Figure 5:**
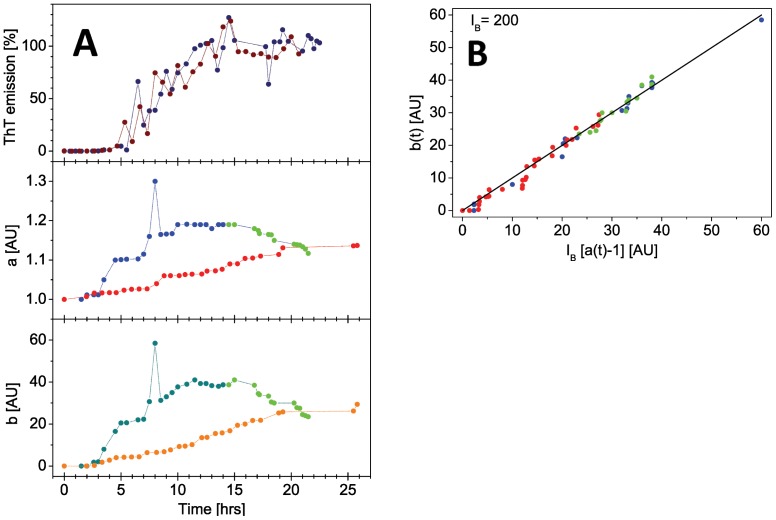
Calculated *a* and *b* parameters. (A): Development in ThT emission (top panel, wt in dark blue, A30P in brown), *a* values (middle panel) and *b* values (bottom panel) are plotted over time for wt (blue and green colours; clear green is used for time-points after the turning point, where the process begins to reverse) and A30P (red and orange colours). ThT intensity is reported on the same timescale for comparison (top). (B): the correlation between the *a* and *b* parameters is plotted. It is evident that the values remain correlated both before (blue) and after (green) the turning point in the development for wt protein scattering. Note that the units for *a* and *b* (though denoted as arbitrary units) are correlated via eq. 3.

The a(t) and b(t) parameters show different behavior for wt αSN versus A30P over time (Fig. 5A): For the wt protein, the a(t) parameter increases during the first 10 h; from approximately 10 until 15 h the value stays constant, and after 15 h it decreases. In the case of A30P, the a(t) parameter only increases linearly in the same time range and thus shows a fundamentally different behavior compared to the wt scattering data. Similar behavior is evident when plotting b(t) values.

In order to further interpret the observed differences, we investigated if a(t) and b(t) are correlated (Fig 5B). Indeed, a and b values correlate for both wt and mutant protein, b(t) = I_B_(a(t)−1) (Fig. 5B). The time-dependent a-value can be related to both the solute concentration and the average relative scattering contrast between solute and solvent, while b is related to the scattering of the solvent alone. The expression can be rewritten to 1/a = [I(t)-I_B_]/[I(0)-I_B_], which demonstrates that a is directly related to variation in the scattering contrast between solute and solvent over time.

Since both b-values and a-values vary over time and are correlated (Fig. 5B), we conclude that there is a change both in the average solvent scattering length density and in the overall contrast between protein and the average solvent. In other words, the observed scattering can no longer be attributed to a two-phase system, composed of protein with a constant average scattering density, and solvent with a constant average scattering density. This suggests one out of two possible explanations: (1) the internal structural changes in protein fibrils are so dramatic, that the mean scattering length density of only a part of this fibril structure is no longer comparable to standard protein structure or (2) the organization of the solvent changes in a part of the solvent into a non-standard solvent behavior. Option (1) is very difficult to imagine in physical terms, hence we favor the option (2). Such a modified solvent layer would most likely be present in the vicinity of the surface of the protein fibrils, hence constitute a significant hydration layer of modified density. Either of the two explanations would result in a three-phase system (solvent, protein and the new third non-standard material phase).

To summarize, we deduce the following from our SAXS analysis. Firstly, for both wt and A30P there are significant structural changes in solution after reaching a steady state ThT fluorescence plateau, Secondly, both a and b values change over time and with a different time dependency for wt and A30P protein. Thirdly, a and b directly correlate for both wt and A30P. Fourthly, the data suggest that either solvent or solute in part changes relative scattering contrast, meaning that the solvent must form a layer around the fibril surfaces that can be distinguished from that of bulk solvent. This is consistent with experimental results and simulations [31].

### Linear Dichroism Reveals Two Phases in the Formation and Reorganization of wt αSN Fibrils

**Figure 6:**
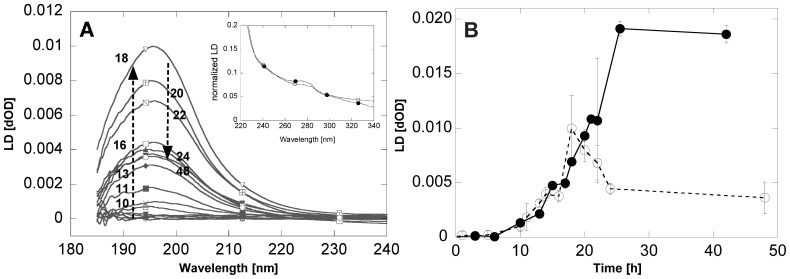
Linear dichroism analysis of aSN fibrillation. (A): LD spectra of wt aSN at different timepoints (hrs of incubation indicated by numbers), showing the peak at 195.6 nm growing over time up to the maximum intensity found after 18 h. Insert: Near-UV spectral region of wt (open symbols) and A30P (filled symbols) aSN show that the tyrosine residues on average are aligned more parallel than perpendicular to the fibre axis. (B): LD data for wt (open symbols) and A30P aSN (filled symbols) as a function time. The data represent the growth of the backbone peak, having λ_max_ at 195.6 nm. Each point represents the average of three replicates and the error bars their standard deviation.

Another technique that allows us to follow the elongation of the fibrils is Linear Dichroism (LD). This technique is critically dependent on fibril length and can monitor the progressive growth of the backbone signal around 195–198 nm [32] as an indicator of cross-β structure being formed, as well as give indications on the lateral interactions between the fibrils which influence the ability of the fibrils to align in the flow. The LD spectra of wt and A30P aSN fibrils show a large peak with a maximum around 195.6 nm (Fig. 6A) attributed to the protein backbone [33], [34]. The peak becomes visible when fibrils are formed and elongate over time (Fig. 6B) in accordance with ThT measurements (Fig. 1). Further, a smaller peak around 275 nm was observed in the near UV region of both wt and A30P (Fig. 6A, insert) at the end of the fibrillation experiment. Since aSN lacks Trp residues, the signal may be attributed to Tyr residues (residues 39, 125, 133, 136) which thus *on average* are aligned more parallel than perpendicular to the fibre axis [32].

After an initial lag phase around 5 h, the LD signal of wt solutions increases up to a maximum around 18 h, corresponding to the final plateau in ThT measurements. However, the backbone LD signals of wt samples then decreases and reaches a new plateau around 24 h onwards, suggesting additional rearrangements of wt fibrils after reaching the ThT plateau. A simpler course was observed in A30P samples where the LD signal continued to rise after the lag phase to a plateau around 24 h.

### TEM Images Reveal Variations in the Types of Late Fibril Networks

**Figure 7:**
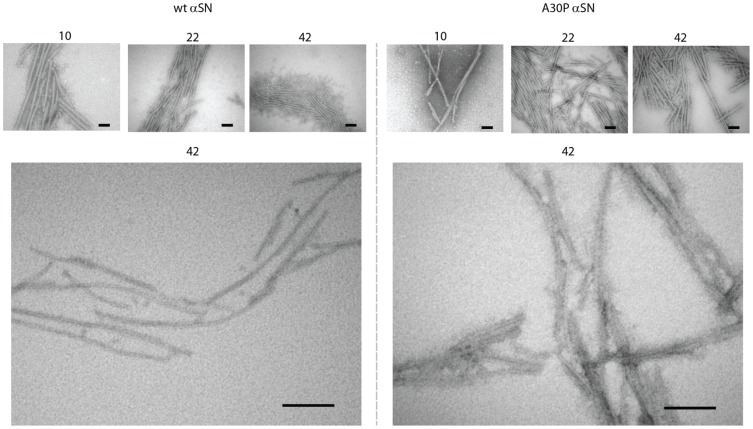
TEM images of wt and A30P aSN fibril samples. Images recorded at specific time points during fibrillation indicated by numbers in the top (hrs). Scale bars are 100 nm.

To obtain direct information on the fibrils’ intermolecular interactions, we acquired TEM images of wt and A30P αSN samples at specific time points. TEM revealed no fibrillar species during the lag phase and did not provide information on oligomer populations (data not shown). However, the representative TEM images in Fig. 7 at time points beyond the lag phase illustrate the fibrils, which emerge and grow as a function of time and are often found associated laterally in fibril networks in both samples. We observed no disruption and breakage of fibrils or shortening of fibril length at later time points for either wt or A30P αSN samples.

At 22–42 hrs of incubation, there is a tendency that wt αSN forms laterally associated clusters over time while A30P forms a denser and more interwoven network of fibrils than wt αSN. This was further confirmed at higher magnifications (Fig. 7, bottom panel) in which A30P fibrils displayed increased propensity to form interwoven fibril networks, and single isolated fibrils were rarely observed in contrast to comparable wt αSN samples.

### X-ray Fibre Diffraction Demonstrates Subtle Structural Differences between wt and A30P that Persist Over Time

**Figure 8:**
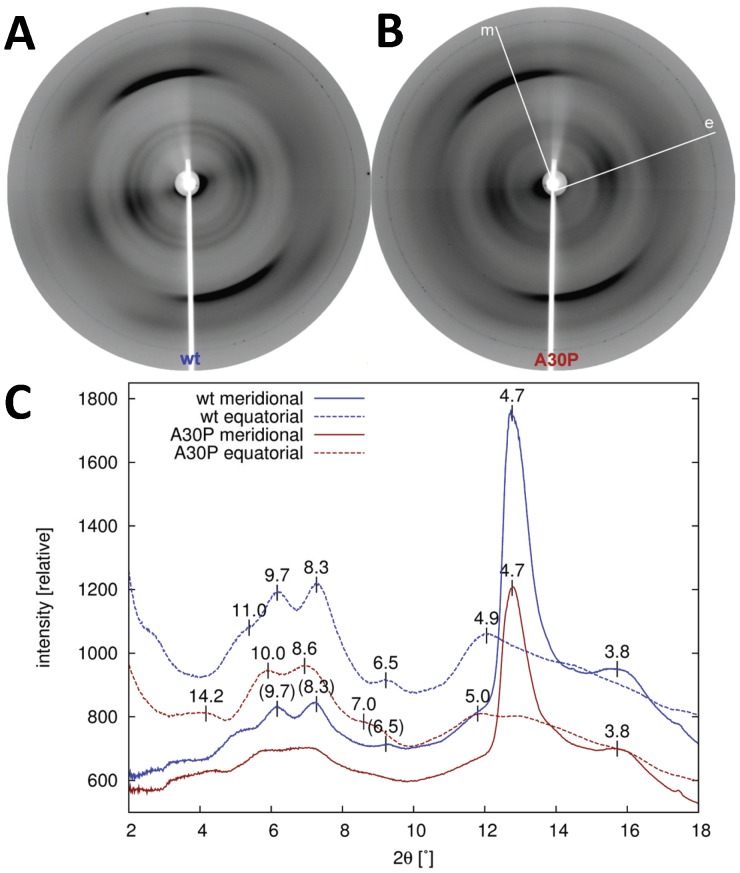
X-ray diffraction patterns of fibrillated protein. Data are for (A) wt αSN and (B) A30P αSN. Panel (C) shows radial averaging of the two patterns in 60° slices around the meridional and equatorial directions, denoted *m* and *e*, respectively in (B). These directions correspond to the fibril long axis and cross-section, respectively. The relative intensity is shown as a function of the estimated diffraction angle, not accounting for a flat panel detector, while the real-space distances (in Å) for the peaks are accurately calculated including flat-panel corrections, and annotated for the significant features. The strongest diffraction corresponds to the meridional 4.7 Å reflection and hence reflects the β-strand distance in sheets along the fibril long axis.

X-ray fibre diffraction (FD) was employed to visualize the presence of β-sheet packing in the fibrils [35], [36]. A typical signature of amyloid-like fibrils is the cross-β pattern, normally interpreted as diffraction resulting from the repeats in paired β-sheets. But also other typically occurring distances within a given fibril are visible if the fibril sample is well ordered and well aligned. The method can hence also pinpoint fingerprints of the molecular arrangements within fibrils in ideal cases. We have obtained very high quality FD images of both wt and A30P αSN fibrils (Fig. 8), revealing a detailed fingerprint of the microscopic structural arrangements of both wt and A30P αSN. The diffraction pattern of both wt and A30P fibrils show the typical cross-β diffraction pattern, with a meridional reflection at 4.7 Å (corresponding to the distances between strands in a β-sheet along the fibril long axis) and equatorial reflections around 10 Å (corresponding to the inter-sheet spacing in amyloids [35, [37]) as previously reported for aSN wt [3]. Importantly, the two reflections around 10 Å (seen as two concentric rings in Fig. 8AB) are shifted by ∼0.3 Å from 8.3 Å and 9.7 Å in wt samples to 8.6 Å and 10.0 Å in A30P samples (Fig. 8C), indicating increased β-sheet pairing distances in A30P fibrils. For both wt and A30P aSN, additional weaker reflections are present, but not at coinciding positions, e.g. 6.5 Å and 11.0 Å equatorial signals in wt aSN, while a more diffuse signal at 14.2 Å is present in the A30P αSN pattern (Fig. 8B). There are thus directly observable differences at the microscopic level between the wt and mutant αSN fibrils, though the structural basis for this remains unclear. We also collected data from fibrils that were harvested at five different timepoints (from 7–144 h, *i.e.* at early and late times on the steady state ThT plateau) but found no differences over time (data not shown) indicating that a single core fibril structure persists throughout the time-course for both proteins, although the two proteins form different internal structures. The distances probed in the fibre diffraction experiment correspond to 3–30 Å and hence only probe the internal arrangement of the fibrils or individual protofibrils. Macroscopic rearrangements (*e.g.* new lateral clustering) will not be visible in FD data. The invariance over time of our FD data suggest that the time-dependent differences that have been observed by LD and SAXS are only related to macroscopic re-arrangements, and not related to microscopic structural changes in the internal protofibril arrangement. We therefore decided to investigate the overall organization interfibrillar arrangement of the fibrils using microscopy.

### Confocal Microscopy Reveals Compact Fibril Networks in A30P but not wt Fibrils

**Figure 9:**
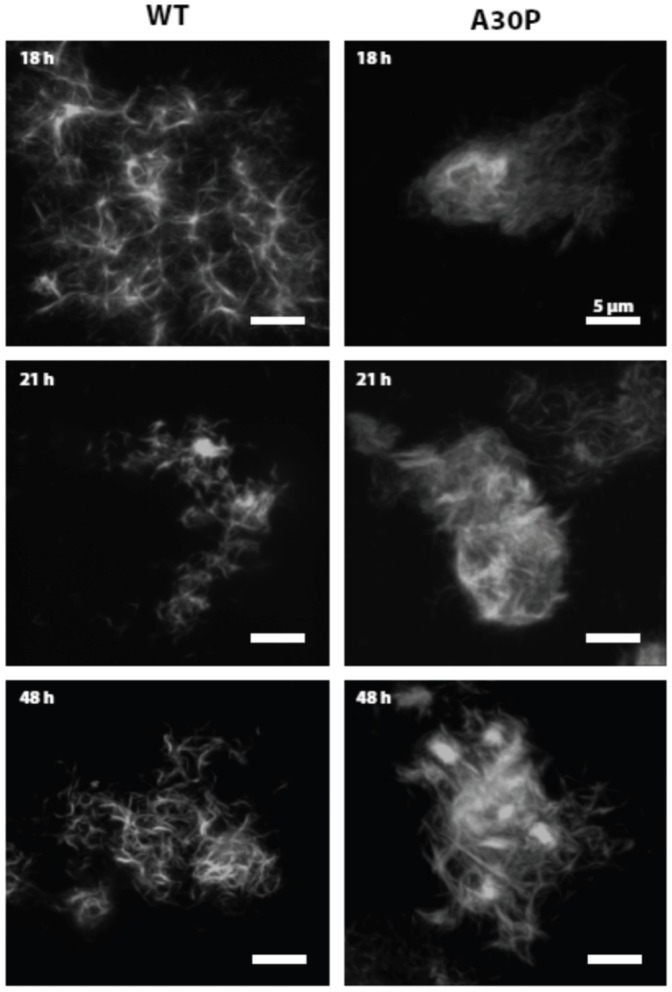
Confocal fluorescence microscopy of αSN fibrils. Images are projections of individual z-stacks for wt and A30P αSN fibrils formed over 2 days, at discrete time steps. There is a marked difference in the fibril network formed by wt and A30P aSN, where the wt form a more open network compared to the apparent stronger interfibril binding for the A30P. Scale bar shows 5 µm for all images. Z-depth is approximately 10 µm in height.

We investigated potential differences in the organization of fibrils at the macroscopic level using confocal fluorescence microscopy (CFM) in which the fibril structure was kept hydrated throughout the image acquisition. Highly detailed Z-stacks were acquired to focus on the macro-structures rather than single fibres confined near the glass surface. Z-stacks projections covering approximately 10 µm (Fig. 9) compare A30P and wt aSN fibres at specific time points during the fibrillation process.

In agreement with the lag phase observed from ThT fluorescence data, no fibrils could be found in solution at 3 h. However, a sparsely interconnected meshwork of what appear to be individual fibrils was observed in wt aSN samples at 18, 21, and 48 h of incubation (although we cannot rule out stacking of two or more individual fibrils due to inherent limitations in CFM resolution). In contrast, the A30P mutant showed a much more densely packed morphology with increased fibril association/bundling at the same time points (Fig. 9).

### NMR Water Relaxation

**Figure 10:**
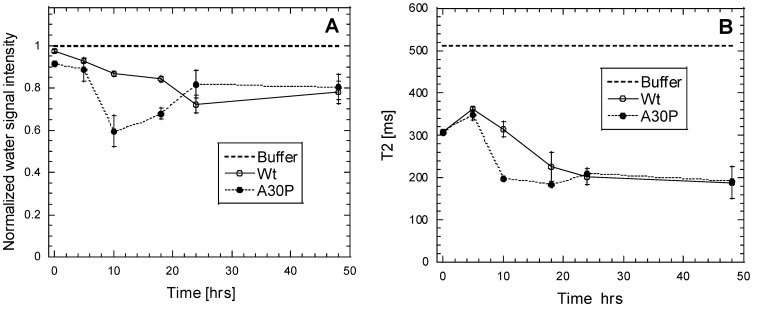
^1^H NMR analysis of water in A30P and wt aSN samples. (A) Normalized water signal intensity over time during fibrillation. (B) T_2_ relaxation times during fibrillation.

Finally, to investigate the water association to fibrils, we applied NMR to evaluate the transverse relaxation of water protons. NMR enables us to differentiate the presence of water being tightly bound to fibrillar structures (invisible to NMR) or present as bulk water, or water in a hydration layer in fast exchance with bulk water (visible to NMR). Consequently, water tightly bound within or on the surface of large fibrillar structures leads to a decrease in the water signal integral during fibril formation. Normalized water signal intensities (Fig. 10A) reveal that A30P αSN incorporates a large amount of water within the first ∼10 hrs of fibril formation followed by a partial release of the water, while wt αSN shows a more steady increase of water incorporation until 20–25 hrs, after which some water is released to bulk. T_2_ relaxation measurements may further differentiate contributions from bulk water with long relaxation time and water influenced by protein, such as hydration water associated to any protein surface (e.g. monomer, oligomers, and fibril) and water trapped in cavities of protein structures all subject to faster relaxation [38]. T_2_ relaxation measurements (Fig. 10B) of the water association to αSN species during the fibrillation course revealed an initial increase in T_2_ relaxation time which is not readily explainable but may be linked to early aggregation effects such as oligomer formation during the lag phase. After 5 hrs of fibrillation, a rapid decrease in T_2_ relaxation time was observed in A30P αSN samples, coinciding with the drop in residual monomer concentration. The T_2_ relaxation time of A30P αSN samples was essentially constant from ∼10 hrs onwards. The T_2_ relaxation time of wt αSN samples displayed a slower initial decrease in the relaxation time, but converged with the relaxation time of A30P samples after ∼18–24 hrs of incubation.

## Discussion

### ThT Fluorescence and Residual Monomer Concentration Highlight Differences between wt and A30P Fibrillation

In this study, we investigated the fibrillation of wt and A30P aSN to understand differences between the two types of fibrils, starting by comparing their kinetic profiles, following the fibrillation by means of ThT fluorescence (∼proportional to length concentration of fibrils) and the residual monomer concentration (Fig 1–2).

Given that the two proteins have almost identical sequences, fibrils of wt aSN and A30P are expected to give rise to similar ThT signal if the fibrils are identical in structure and amount. However, our data consistently showed that A30P aSN give rise to significantly lower ThT intensity (∼30% of the wildtype ThT intensity) throughout the fibrillation (Fig. 1), although the amount of fibrils produced upon completion of the fibrillation process is essentially the same, as shown by the residual soluble aSN concentration (Fig. 2). We have no reason to believe that this difference can be attributed to variability in the fibrillation assay. Our fibrillation conditions are optimized for reproducibility through a combination of plate reader shaking and inclusion of glass beads [10]. Furthermore, the differences between the two proteins are reproduced using other techniques. The significant difference in ThT intensity immediately suggests differences in fibril structure or physicochemical properties of interacting surfaces which in both cases ultimately lead to different modes of binding of ThT. Indeed, a more polar and/or hydrated microenvironment surrounding A30P fibrils has been reported [39], again suggesting slightly altered binding site(s) or structural changes which allows water to penetrate further into the structure and quench the ThT fluorescence. Similar observations have been made for A30P and wt aSN fibrils grown at 25°C (A30P aSN intensity ∼25% that of wt aSN at apparent fibrillation endpoint) [40] and for the peptide hormone glucagon, which – depending on solvent conditions - forms different fibrils with very different ThT binding properties [41].

The decline in residual soluble species followed closely the ThT signals as shown by the initial lag phase of ∼5 h and plateau from ∼18 h onward. However, the slightly steeper decrease in residual soluble A30P species from 5–10 h (significantly different from wt when taking the very low standard errors into account) suggests a slightly more rapid initial aggregation rate but a slightly slower final aggregation rate for A30P. We note that wt aSN fibrillates through a nucleation-dependent polymerization mechanism [42] and that the kinetics of A30P (and A53T) aSN has been shown to be faster than the wildtype [7] at concentrations well below the supercritical concentration (SCC). However, in this study we have fibrillated aSN above the SCC and this may explain the similar aggregation kinetics of the two αSN mutants through a shift in the monomer-oligomer equilibrium at such protein concentration [11].

### SAXS Analyses Indicate that the Solvent-fibril Interactions Change during the Fibrillation Process in Different Ways for wt and A30P αSN

Our SAXS data confirmed qualitative and quantitative differences between the structural development in solution for A30P and wt aSN. Firstly, the fibrils continue to evolve their structure after reaching the ThT plateau as evidenced by the continued development at low s values (Fig. 3B). This was already published for wt aSN [22] and we confirm this qualitative behavior for A30P, although the SAXS data cannot be used to reconstruct structural models of these changes. Within the current experiment, it has not been possible to obtain data from adequately homogeneous mature fibrils. This complexity rules out decomposition of data curves and *ab initio* modeling from the reconstructed data from isolated species. Secondly, a surprising development of the scattering signal at higher angles is observed, which clearly differs for the two protein systems. While the background buffer measurements remain stable, the protein scattering reveals systematic changes, which hence reflect a change in the sample composition. This change can be quantified (see Fig. 5) as described in Results. Such a change could, as a first intuitive interpretation, arise if the protein fibrils were precipitating extensively from solution, which would result in a lower overall protein concentration in the sample exposed to scattering. However, we rule out this scenario as we did not observe any precipitation during the scattering experiment. Even low concentrations of protein samples would never result in a scattering signal of lower intensity than the pure solvent scattering signal. Further, when analyzing the deviations in scattering at high angles, the changes are not only concentration dependent. If there was only a strict concentration dependency, the development should be described only by a multiplication factor (changes in a-values) since scattering is proportional to concentration. In contrast, we see that both a- and b-values vary with time. Interestingly, the observed changes reverse in wt protein where the a- and b-values start decreasing after 15–20 h. In contrast, the development continues within the observed time ranges for A30P, indicating a continuous structural development throughout the timespan.

Furthermore, we observe that a- and b-values correlate, and our analysis reveals that the time-dependent development of a- and b-values differs. The correlation, as described in the Results section, allows us to conclude that the variation of the contrast is due to a system that is more complex than a simple two-phase system. In our view this is best interpreted as a system, where solvent has formed significant hydration layers and/or is clearly organized with respect to the fibrils. This is qualitatively evident in the raw SAXS curves, where the slope at high s-values decreases as a function of the time. Consistent with this, Huang and colleagues have also reported variation in water structure at increasing temperature [29] which demonstrates that water structure is a time and temperature dynamic feature. It is also evident from the time-invariant FD data that there are no internal structural changes in the fibrils over time which otherwise could have given rise to *e.g.* a change in the mean scattering density of the protein fibril structure itself. This leaves the possibility that a layer of water is organized in a non-bulk fashion in vicinity of the fibril surfaces, and this layer has a contrast to both the protein-part of this macroscopic fibril structure, and to bulk water [31]. Obviously, if the fibrils align or cluster laterally, this will further influence the organization of such a water layer. Tight protein:protein associations in general minimize the surface area available for forming a hydration layer. We have observed that the hydration effects increase over time for A30P, while they appear to decrease over time for wt fibrils (Fig. 5). Thus, either the changes are reversible or there are two different phenomena evolving at the same time, one causing an increase, the other causing a decrease in high-angle scattering.

While our FD data do not reveal any structural changes over time, they do clearly point out that there are internal structural differences between the wt and mutant fibrils. The systematically repeating features in the fibrils hence are different between the two types of fibrils. This is in accordance with previous observations, based on atomic force microscopy analysis [43], [44] which also have reported structural differences between wt and mutant aSN fibrils. In these previous studies, variations in periodicity and protofilament width and assembly were observed (i.e. at the nm scale). Rather, here, we reveal that the differences between the mutant and wt fibrils propagate based on differences at the detailed atomic level (e.g. the presence of a 6.5 Å and 14.2 Å signal for the mutant fibrils, with a different repeating distance around 11 Å is revealed for wt fibrils).

### TEM and CFM Images Suggest that the SAXS Changes are Related to Different Levels of Lateral Fibril Association, which may Affect Solution Viscosity

Such differences in the macroscopic arrangement are elaborated by our TEM and CFM images. These techniques reveal more dense fibril networks for A30P, whereas wt fibrils have an increased tendency to associate laterally. From the scattering data, it seems that these macroscopic structural changes influence the solvent behavior significantly. The initial increase in a- and b-values suggests a change in the degree of structured water. The reversed effect observed for wt protein at later time points hence points to an effect of the macroscopic changes, again changing the degree of layered water on fibril surfaces. The continued increase in a- and b-values is correlated with the formation of very dense networks (as observed by microscopy methods) and hence this lateral association is suggested to further increase the effect observed in scattering. Microscopy results did not clearly reveal why the effect is reversed in wt aSN fibrils. The reversed effect is however confirmed in LD results.

We therefore conclude that the two types of protein fibrils may cause different effects on the solvent, and this seems to be related to macroscopic changes, rather than microscopic changes in the internal protein structures. The interesting observation that there are differences in such effects for the wt and familial mutant proteins raise the question whether such variations in the effect on the fibril environment also exist *in vivo*, but this is outside the scope of the current investigation. To our knowledge there are no techniques that have addressed possible changes in wt and A30P αSN fibril arrangements *in vivo* at the current level of detail.

Although the LD data are recorded at a lower overall protein concentration, they confirm the time dependent behavior. There is an approximately sigmoidal LD signal development for A30P aSN after reaching the ThT plateau, while wt aSN showed maximum LD intensity after 18 h of fibrillation, followed by a drop to a plateau of lower intensity. Both LD and SAXS thus show that the structural development of wt aSN passes through a maximum in terms of LD signal, while A30P undergoes a steady increase. Moreover, the LD data confirm the presence of continued structural development or rearrangements of the A30P mutant after reaching the ThT plateau, although the same amount of fibril mass is obtained in both cases (Fig. 2).

The LD signal decay observed in wt aSN after 18 hours (Fig. 6B) is likely caused by a reduction of the degree of alignment of the fibrils in the laminar flow. This may be caused by either significant fragmentation of the fibrils or by the formation of extensive networks between the fibrils, which deviate from the ideal rod-shape and thus effectively reduce their ability to align in the laminar flow. Fibril breakage/fragmentation was not observed in TEM images and no increase in soluble species at later stages in the fibrillation was observed from residual aSN concentration measurements. However, an increased tendency towards lateral fibril association was observed in TEM images of wt samples from 22–42 hrs, which may explain the decrease in LD signals.

### NMR Measurements Reveals Difference in Water-association to A30P and wt aSN Samples

Wt aSN samples showed a slower decrease in the NMR water signal than A30P (Fig. 10A), in agreement with tightly packed, laterally associated fibrils with little room for bound water, as compared to isolated fibrils. In contrast, the faster association of water to A30P species (0–9 h) may arise partly from more water being trapped in the larger sheet spacing of these fibrils and partly as tightly bound surface water. The following increase in the water signal from A30P samples (∼9–24 h) until the plateau around 24 hrs may be explained by exclusion of water from fibril structures at later stages during the formation of increasingly dense fibrils networks also demonstrated in CFM images.

The dense fibrillar networks observed by CFM and TEM in A30P samples may be more susceptible to shear flow forces, allowing individual fibrils to align. Simple manual aspiration of samples indicated that the viscosity of wt aSN approximately follows the course of LD signals, namely an increase in viscosity around 18 h followed by a decrease around 144 h. Conversely, an increasingly thicker gel was formed in A30P aSN from approximately 10 h of fibrillation onwards devoid of the “thinning” phenomena observed for wt aSN. This suggests a difference in the degree and/or mode of fibril association in which A30P aSN forms could form more disordered and extended fibril networks than wt aSN. TEM images indicated that wt αSN formed most extensive fibril networks at intermediate time points, followed by the formation of more “constrained” bundles of laterally associated fibrils at later stages (Fig. 7), which is consistent with the changes in LD signals and viscosity. TEM also gave tentative indications that A30P fibrils had a tendency to bundle and intertwine to a greater extent than wt αSN (Fig. 7). This was reinforced by confocal laser microscopy which revealed that A30P fibrils consistently displayed a more extensive and densely packed macroscopic fibril arrangement than wt aSN samples (Fig. 9). This could indicate a stronger inter-fibril binding or increased number of contact points between A30P aSN fibrils and is likely the reason for the observed increased in sample viscosity. The complex relationships between fibril structure, bundling, intertwining and networking makes it difficult to make simple predictions about the relationship between altered clustering and altered LD signals, but clearly changes in fibril organization will affect the LD signal, and our results are consistent with this.

### Summary

In conclusion, we have complementary observations from a number of methods, which in different ways suggest that the solvent behavior during protein fibrillation is more complex than previously anticipated. We suggest that a dense solvent phase is formed in the vicinity of the fibril surface, and that the extent of this phase is so significant that it is clearly observable both by the decay in NMR bulk water signal and through the high angle X-ray scattering signal. Such a modified solvent phase in the vicinity of fibril surface likely influences fibril macroscopic features (bundling, network formation, overall fibril morphology). This is indeed observed in our TEM analysis. On a more speculative note, such a modified surface layer could play a significant role in vivo since altered surface properties and altered overall morphology would also result in varied interactions with other cellular components such as the membrane, organelles, the proteasome, etc.
